# Clinical Characteristics and Outcomes of Incomplete Cardia Reconstruction Cases Following Multiple Anti‐reflux Mucosectomy/Anti‐reflux Mucosal Ablation Treatments

**DOI:** 10.1002/deo2.70169

**Published:** 2025-07-01

**Authors:** Ippei Tanaka, Haruhiro Inoue, Mayo Tanabe, Yoshiaki Kimoto, Kei Ushikubo, Kazuki Yamamoto, Yohei Nishikawa, Nikko Theodore Valencia Raymundo, Kazuya Sumi

**Affiliations:** ^1^ Digestive Diseases Center Showa University Koto Toyosu Hospital Tokyo Japan

**Keywords:** anti‐reflux mucosal ablation (ARMA), anti‐reflux mucosal intervention, anti‐reflux mucosectomy (ARMS), endoscopic treatment, gastroesophageal reflux disease

## Abstract

**Introduction:**

Anti‐reflux mucosectomy (ARMS) and anti‐reflux mucosal ablation (ARMA) are novel endoscopic treatments for proton pump inhibitor or potassium‐competitive acid blocker‐refractory gastroesophageal reflux disease. These procedures induce scarring of the artificial ulcer at the gastric cardia, which tightens the enlarged cardiac opening. However, we encountered patients with unresolved symptoms due to insufficient cardiac shrinkage despite multiple ARMS/ARMA treatments. This study analyzed the frequency and characteristics of the refractory cases.

**Methods:**

We retrospectively reviewed patients who underwent ARMS/ARMA treatments at our institution from January 2014 to October 2022. Refractory cases were defined as those undergoing multiple ARMS/ARMA treatments but with insufficient cardiac shrinkage and also persistence of reflux symptoms.

**Results:**

Out of 131 ARMS/ARMA patients, seven (5.3%) cases were categorized as refractory cases. The male‐to‐female ratio was 5:2, with a mean age of 70 (58–73) years, and a mean BMI of 20.4 (20.0–24.0). Gastroesophageal reflux disease grades were grade *N* (*n* = 2), M (*n* = 2), B (*n* = 2), and C (*n* = 1). All had NERD diagnosis confirmed by pH monitoring. One patient underwent ARMS twice, two underwent ARMA after ARMS, and four had ARMA more than twice, but none achieved sufficient cardia shrinkage. Finally, three cases were managed with medication, while four required surgical fundoplication, which improved their symptoms.

**Conclusion:**

Approximately 5% of ARMS/ARMA cases could not achieve sufficient cardiac shrinkage. However, the surgical treatment performed as a final step was effective, which may suggest that ARMS/ARMA treatment serves as a treatment option between medical therapy and surgical treatment.

## 1 Introduction

The global prevalence of gastroesophageal reflux disease (GERD) is estimated at 14.8%, with its incidence continuing to rise [1, 2]. In Japan, approximately 15% of the population experience reflux symptoms weekly, making it one of the most common upper gastrointestinal tract diseases diagnosed in routine clinical practice [3]. The primary treatment options for GERD are oral administration of Proton Pump Inhibitors (PPIs) and potassium‐competitive acid blockers (P‐CABs) [4]; however, 40% of patients exhibit resistance or dependence on these oral modalities [5].

As a treatment for such patients, Inoue et al. performed antireflux mucosectomy (ARMS) and published the first case series of patients treated with ARMS in 2014 [6]. ARMS is an endoscopic procedure involving the resection of the mucosa around the cardia, which creates an artificial ulcer. Subsequent scar formation during the healing process results in narrowing of the cardiac opening, leading to favorable outcomes in GERD symptoms. In 2020, a simplified method of ARMS called antireflux mucosal ablation (ARMA) was proposed, which utilizes mucosal ablation through coagulation current or argon plasma coagulation (APC) instead of mucosal resection [7]. This method achieved clinical outcomes comparable to those of ARMS. Furthermore, the long‐term outcomes of both ARMS and ARMA have also been confirmed to be effective [8, 9], and their safety and efficacy have also been demonstrated in systematic reviews and meta‐analyses [10, 11, 12]. At our hospital, these treatments have been performed for nearly 10 years since 2011, and we have encountered cases with insufficient cardiac shrinkage even after multiple ARMS or ARMA treatments. To date, there has been no research on such refractory cases. Therefore, the aim of this study is to identify the frequency and characteristics of refractory cases.

## 2 Method

### 2.1 Study Design

This study was a retrospective study conducted at Showa University Koto Toyosu Hospital. Data on patients who underwent ARMS or ARMA between January 2014 and October 2022 for PPI‐refractory or PPI‐dependent GERD were extracted. Data regarding the patients' physical characteristics, symptoms, and treatment‐related information were obtained from electronic medical records. The Institutional Review Board of Showa University approved the study (Approval numbers: C‐T2025‐1108). All patients involved in the study provided written informed consent for the treatment procedure.

### 2.2 Case Selection

Inclusion criteria in this study were refractory cases, which was defined as follows; (1) patients who underwent ARMS or ARMA treatments multiple times, (2) cases that experienced insufficient shrinkage of the cardia after endoscopic treatment, which was defined as both contraction rate of the cardia by less than 50% and Hill's grade II or higher based on the Hill's Classification of the gastroesophageal flap valve (GEFV) [13], (3) cases which showed no improvement in symptoms. Symptoms before and after treatment were objectively assessed using patient‐reported tools, including the GerdQ [14] and the Frequency Scale for the Symptoms of GERD (FSSG) questionnaires [15]. The exclusion criteria included cases where the ulcer was sutured and cases without available imaging or incomplete data. After identifying refractory cases, we collected data on the patient's age, gender, body mass index (BMI), American Society of Anesthesiologists Physical Status (ASA‐PS) classification, prior GERD treatments, GERD classifications (Los Angeles [LA] classification) [16], presence of atrophy, and clinical course.

#### 2.2.1 Patient Selection

Patients eligible for ARMS/ARMA treatments were those who, despite taking a standard dose of PPI for more than 8 weeks or 20 mg of *P‐CABs* for more than 4 weeks, continued to experience typical reflux symptoms at least twice a week, indicating PPI‐refractory or PPI‐dependent GERD. Upper gastrointestinal endoscopy, high‐resolution manometry (HRM; Star Medical Inc., Tokyo, Japan), esophagography with barium, and biopsy when necessary were performed. GERD diagnosis was made based on endoscopic findings and 24‐hour pH monitoring (acid exposure time [AET], DeMeester composite score, symptom index [SI], and symptom association probability [SAP]). Patients who were diagnosed with esophageal motility disorders, eosinophilic esophagitis, large hiatal hernias, and functional heartburn were excluded [7, 17].

#### 2.2.2 ARMS and ARMA Procedures

ARMS was performed by a single operator (Haruhiro Inoue), using a cap‐fitted endoscopic mucosal resection (EMR‐C) technique with a Q260J endoscope (Olympus Corp., Tokyo, Japan). A large, hard cap with an oblique cut (MAJ‐296; Olympus) was used together with a crescent snare (SD‐221L‐25; Olympus). Saline mixed with indigo carmine was injected into the submucosa to facilitate mucosal lifting, and multiple resections using the EMR‐C technique were performed, primarily along the lesser curvature of the cardia, resulting in the removal of approximately two‐thirds to four‐fifths of the circumference. The resection of esophageal mucosa was avoided, as it may lead to stenosis. Furthermore, since August 2017, with the aim of preventing transient stenosis, we have preserved a small portion of mucosa in the lesser curvature in a butterfly‐like shape. Thus, the original resection method was adopted in the first 63 patients, while the butterfly method was adopted in the latter 20 patients.

Similarly, ARMA procedures were performed by a single operator (Haruhiro Inoue) using the same gastroscope as ARMS, with a super‐soft hood (Space Adjuster; TOP, Tokyo, Japan) and triangle‐tip knife J (TTJ; Olympus, Tokyo). Saline with indigo carmine was injected into the submucosa to reduce thermal injury during the procedure. Mucosal ablation was performed in a butterfly shape around the cardia, similar to the ARMS procedure.

### 2.3 Postoperative Management and Follow‐up

PPI therapy was continued for 4 weeks after both ARMS and ARMA. Follow‐up endoscopy was scheduled approximately one to three months after the procedure, during which questionnaire responses were collected. If sufficient shrinkage of the cardiac opening was observed, along with improvement in symptom scores, annual follow‐up was planned. On the other hand, if these findings were not observed, outpatient follow‐up every 2–3 months was continued.

## 3 Result

**FIGURE 1:**
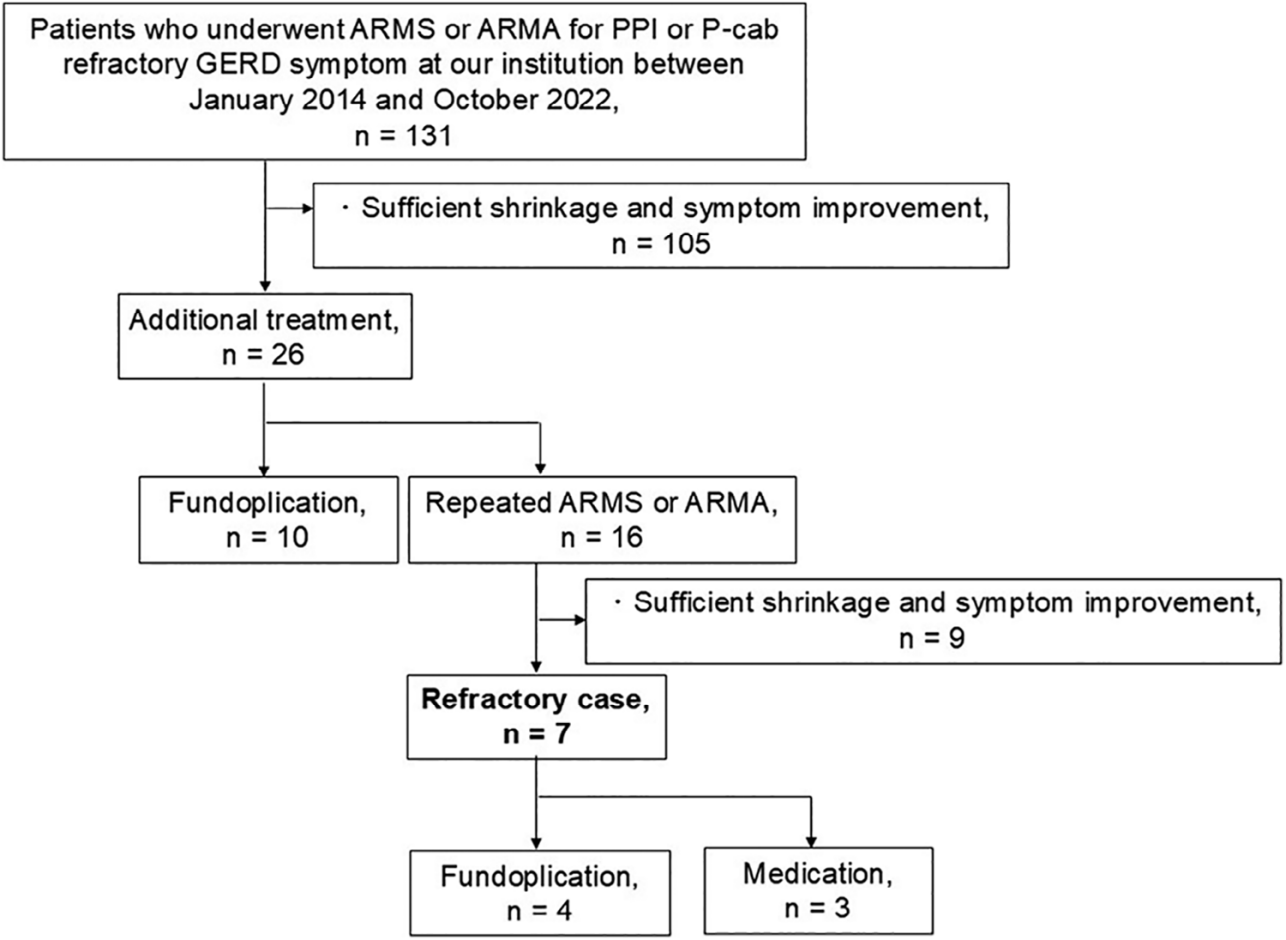
Flow diagram of the enrolled patients in this study. ARMS, antireflux mucosectomy; ARMA, antireflux mucosal ablation; PPI, proton pump inhibitor; P‐cab, potassium‐competitive acid blocker; GERD, gastroesophageal reflux disease.

**FIGURE 2:**
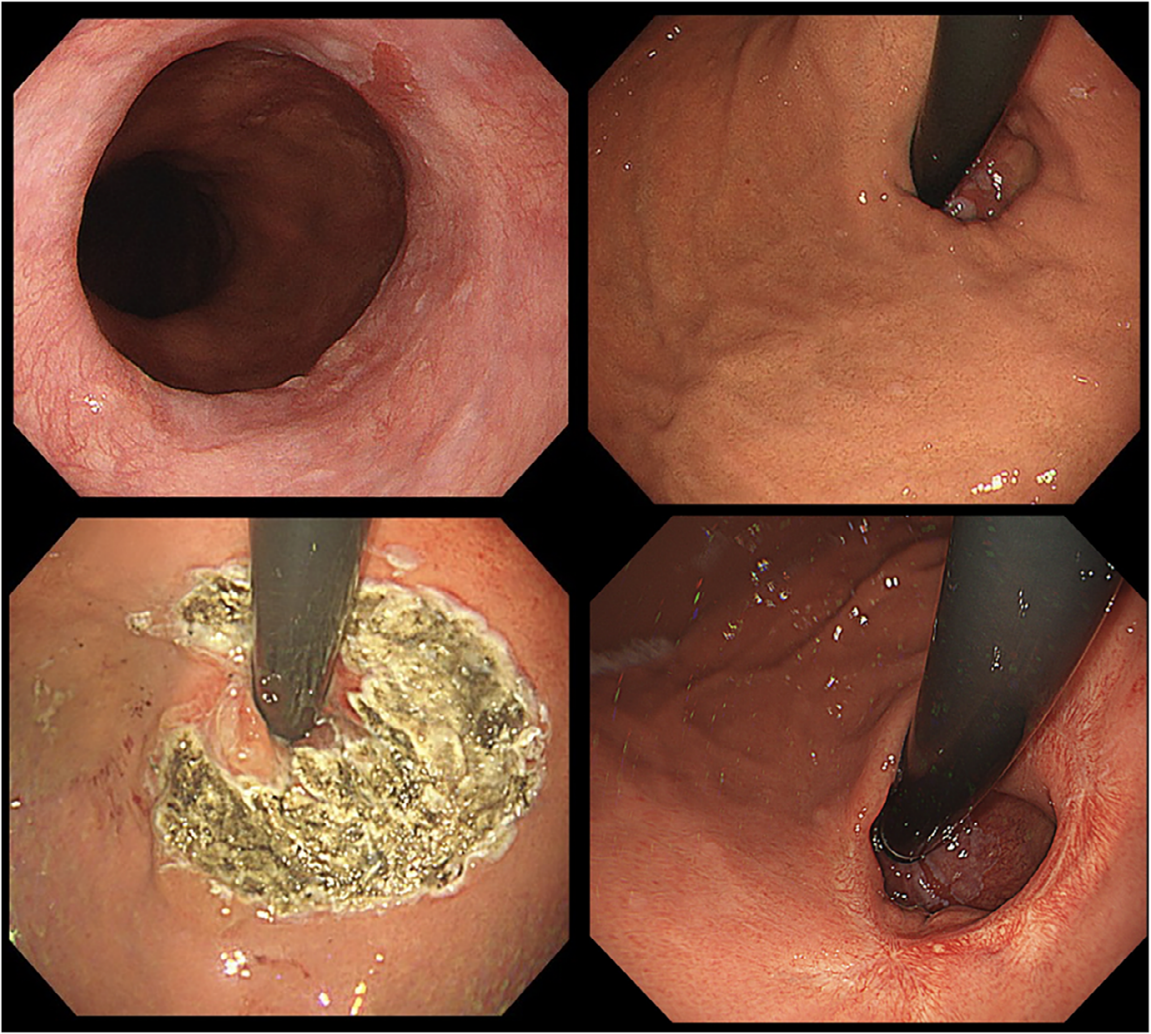
Endoscopic images of the cardia before and during the first ARMA procedure. (Case 4). (A and B) Prior to the first ARMA. Endoscopic image in retroflex position demonstrated significant valve opening, consistent with a Hill grade III flap valve. (C) Immediately after the first ARMA. (D) One month after the first ARMA. This image shows inadequate shrinkage of the cardia. ARMA, antireflux mucosal ablation.

**FIGURE 3:**
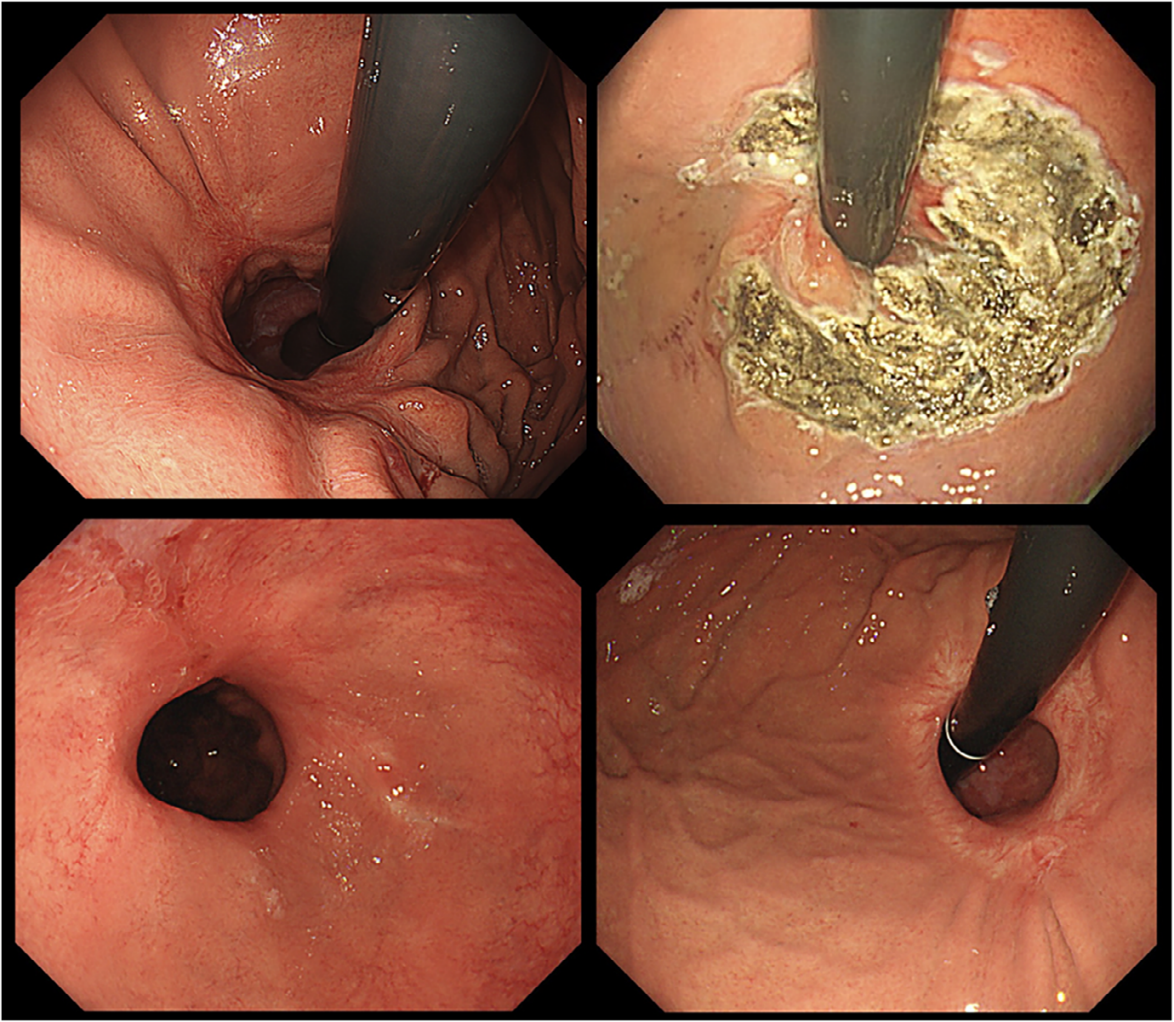
Endoscopic images after the first ARMA treatment and after six repeated ARMA procedures. (Case 4). (A) After the first ARMA. Endoscopic image in retroflex position demonstrated significant valve opening, consistent with a Hill grade III flap valve. (B) Immediately after the second ARMA. (C and D) One month after the 6th ARMA. These images show inadequate shrinkage of the cardia. ARMA, antireflux mucosal ablation.

A flow diagram of the patients in this study is shown in Figure 1. Among the 131 patients who underwent ARMS or ARMA treatment at our institution between January 2014 and October 2022, 26 cases (19.8%) exhibited insufficient shrinkage after a single treatment. Ten patients underwent surgical hernia repair procedures, such as Nissen or Toupet fundoplication, while sixteen cases (12.2%) received additional endoscopic treatment. Among those who underwent two or more endoscopic procedures, nine patients achieved sufficient shrinkage of the cardia and symptom improvement; however, seven cases (5.3%) remained refractory; hence, were classified as refractory cases. Representative images of refractory cases are shown in Figure 2 and Figure 3.

Among the refractory cases, the male‐to‐female ratio was 5:2, with a mean age of 70 years (range, 49–75) and a mean BMI of 20.4 (range, 18.0–25.4). ASA scores were one in five patients and two in two patients. Two patients were on antithrombotic medications. Two patients had an LA grade N, two patients had an LA grade M, two patients had an LA grade B, and one patient had an LA grade C. Five patients were classified as Hill's grade II, while two patients were Hill's grade III. No cases of atrophic gastritis were observed. The pH monitoring confirmed that all cases were diagnosed with non‐erosive reflux disease (NERD). Characteristics of the refractory and non‐refractory cases are shown in Table 1.

**TABLE 1:** Characteristics of refractory and non‐refractory cases.

No.	Age (years)	Sex	BMI, kg/m^2^	ASA‐PS	Past medical history	Anticoagulant	NSAIDs or Steroids	PPI refractory or dependent GERD	Los Angeles classification	Atrophic gastritis	Barrett esophagus	Flap valve grade	24‐PH diagnosis	FSSG score pre‐treatment	Total number of ER	Clinical course
1	68	Female	20	2	Postoperative lung cancer, Cerebral infarction	Aspirin	–	Refractory	Grade B	None	SSBE	3	Pure GERD	22	2	ARMS (2)→Fundoplication
2	64	Male	25	1	–	–	–	Refractory	Grade N	None	SSBE	2	Pure GERD	31	2	ARMS→ARMA→Fundoplication
3	66	Male	22	1	–	–	–	Refractory	Grade N	None	–	2	Pure GERD	24	2	ARMS→ARMA→Fundoplication
4	58	Male	25	1	–	–	–	Refractory	Grade M	None	–	2	Pure GERD	13	5	ARMA (5)→Medication
5	75	Female	18	1	–	–	–	Refractory	Grade M	None	–	2	Pure GERD	25	3	ARMA (3)→Fundoplication
6	49	Male	20	1	–	–	–	Refractory	Grade B	None	SSBE	3	Pure GERD	25	3	ARMA (3)→Medication
7	73	Male	20	2	–	Aspirin	–	Dependent	Grade C	None	SSBE	2	Pure GERD	24	2	ARMA (2)→Medication
Non‐ refractory cases (*n* = 1 14)^※^	53 (23–89)	Male 78 (8.4%)	21.3 (14.2–35.7)	1 (1–3)	Other organ cancers: 6, post‐gastrectomy: 4, respiratory diseases: 3, and cardiovascular diseases: 4	Aspirin: 2 (1.8%)	2 (1.8%)	Refractory case 98 (86.0%), Dependent: 16 (14.0%)	Grade B or more 13 (11.4%)	Present 9 (7.9%)	SSBE: 60 (48.3%), LSBE 5 (4.0%)	2 (2–3)	Pure GERD: 76 (52.6%) Reflux Hypersensitivity: 38 (.33.3%)	25 (9–46)	–	–

^※^ Non‐refractory case refers to a case in which reconstruction of the cardia was successfully achieved and symptoms improved after one or multiple ARMS/ARMA treatments. For non‐refractory cases, continuous variables are summarized as medians with ranges, while nominal and ordinal variables are summarized as percentages.

Abbreviations: ARMA, antireflux mucosal ablation; ARMS, antireflux mucosectomy; ASA‐PS, American Society of Anesthesiologists Physical Status; BMI, body mass index; FSSG, frequency scale for the symptoms of GERD; GERD, gastroesophageal reflux disease; LSBE, long segment Barrett's esophagus; NSAIDs, non‐steroidal anti‐inflammatory drugs; PPI, proton pump inhibitor; SSBE, short segment Barrett's esophagus.

One patient underwent ARMS twice, two patients underwent ARMA after ARMS, and four patients underwent ARMA more than twice; however, none achieved sufficient shrinkage of the cardia. Out of the three patients who underwent ARMS treatment first, one patient was treated using the original method, while the remaining two patients underwent the butterfly method. After multiple treatments, only one patient's Hill's grade improved from III to II, while the other six patients showed almost no change from their pre‐treatment Hill's grades. Finally, three cases were managed with medications, including PPI or P‐CAB and gastric prokinetic agents, resulting in relatively stable symptoms. On the other hand, four cases underwent surgical fundoplication, which led to symptom improvement.

## 4 Discussion

In this study, we analyzed treatment‐refractory cases in which the shrinkage of the cardia and symptom improvement remained insufficient despite multiple ARMS or ARMA procedures. A total of seven refractory cases were identified, accounting for 5.3% of all cases. Compared with non‐refractory cases, no specific background characteristics or common factors in refractory cases were identified. At present, predicting which cases may be treatment‐refractory remains challenging. Thus, if sufficient improvement is not achieved after two or three endoscopic treatments, surgical intervention should be considered.

One of the advantages of endoscopic treatment for GERD, such as ARMS and ARMA, is the ability to be performed repeatedly. Upon reviewing the endoscopic images of the seven refractory cases, all cases showed a mild shrinkage of the cardia with each treatment session, although none achieved a flap valve grade I. While symptom improvement was not sufficient, slight improvements were also observed, which may have led to additional treatments primarily based on strong patient preference. Among the sixteen cases (12.2%) that underwent additional treatments, nine showed sufficient improvement, suggesting that repeated endoscopic retreatment, particularly ARMA, is effective. Since ARMA involves only mucosal ablation, which is technically simple to perform, further treatment is feasible when prior endoscopic treatments have resulted in scarring around the cardia [7]. When categorizing cases in which multiple treatments ultimately led to sufficient shrinkage and symptom improvement as successful, 114 out of 131 cases (87.0%) were defined as successful, indicating a high success rate. Given that endoscopic treatment is a minimally invasive approach and includes low complication rates [6, 7, 8, 9, 10, 11, 12], repeated procedures are considered acceptable. Therefore, further validation in a larger cohort may be necessary to confirm the effectiveness of multiple endoscopic treatments.

Twenty‐six cases exhibited insufficient shrinkage after a single treatment, among which 10 patients ultimately underwent surgical hernia repair, such as Nissen or Toupet fundoplication. Additionally, among the seven refractory cases, four patients eventually underwent surgical intervention. At our institution, surgical treatment is generally indicated for cases with an esophageal hiatal hernia measuring 3 cm or larger. However, when surgery is chosen as an additional treatment for PPI‐refractory GERD, we think that further endoscopic treatment is unlikely to provide additional benefit for the patient. Among the patients who underwent surgical treatment in this study, 13 out of 15 (86.7%) experienced symptom improvement. In fact, surgical intervention has established itself as the gold standard treatment for PPI‐refractory GERD [18, 19]. Considering the invasiveness of each treatment approach, it is reasonable to first perform endoscopic treatment for patients with PPI‐refractory GERD, and fundoplication should be considered as the final treatment option, when necessary.

This study has several limitations. First, it was a single‐center, retrospective study. Second, all the cases in this study were performed by expert endoscopists. Therefore, larger multi‐center studies may be needed to examine its generalizability. Third, there are no clear criteria for decision‐making regarding whether to choose endoscopic retreatment or surgical intervention, as the patient's preference also plays a key role in the selection process. Establishing a clearer flowchart in treatment selection would be desirable in the future. Lastly, this study focused exclusively on cases treated with ARMS or ARMA, and did not include those treated with ARM‐P (anti‐reflux mucoplasty) or ARM‐PV (anti‐reflux mucoplasty with valve), which involve closure of the artificial ulcer after mucosal resection by using clips, with or without valve formation. Since ARM‐P and ARM‐PV enable intraoperative control of cardia tightness, no cases of postoperative stenosis have been observed. Therefore, these techniques may also be more effective in preventing refractory cases; however, further studies are needed to confirm this possibility.

In conclusion, approximately 5.3% of ARMS/ARMA cases failed to achieve sufficient shrinkage of the cardia despite repeated endoscopic treatments. No distinct background characteristics or shared factors were found among these refractory cases. Surgical intervention ultimately led to symptom improvement, indicating that endoscopic therapy could serve as an intermediate treatment option between pharmacologic management and surgery.

## Conflicts of Interest

The authors declare no conflicts of interest.

## Ethics Statement

Approval of the research protocol by an Institutional Review Board. Approval number: C‐T2025‐1108

## Consent

All patients involved in the study provided written informed consent for the treatment procedure.

## Clinical Trial Registration

N/A.
